# Identification of plasminogen-binding sites in *Streptococcus suis* enolase that contribute to bacterial translocation across the blood-brain barrier

**DOI:** 10.3389/fcimb.2024.1356628

**Published:** 2024-02-22

**Authors:** Tiantong Zhao, Alex Gussak, Bart van der Hee, Sylvia Brugman, Peter van Baarlen, Jerry M. Wells

**Affiliations:** Host-Microbe Interactomics, Wageningen University & Research, Wageningen, Netherlands

**Keywords:** *Streptococcus suis*, blood-brain barrier, enolase, plasmin(ogen), extracellular matrix, CRISPR/Cas9

## Abstract

*Streptococcus suis* is an emerging zoonotic pathogen that can cause invasive disease commonly associated with meningitis in pigs and humans. To cause meningitis, *S. suis* must cross the blood-brain barrier (BBB) comprising blood vessels that vascularize the central nervous system (CNS). The BBB is highly selective due to interactions with other cell types in the brain and the composition of the extracellular matrix (ECM). Purified streptococcal surface enolase, an essential enzyme participating in glycolysis, can bind human plasminogen (Plg) and plasmin (Pln). Plg has been proposed to increase bacterial traversal across the BBB via conversion to Pln, a protease which cleaves host proteins in the ECM and monocyte chemoattractant protein 1 (MCP1) to disrupt tight junctions. The essentiality of enolase has made it challenging to unequivocally demonstrate its role in binding Plg/Pln on the bacterial surface and confirm its predicted role in facilitating translocation of the BBB. Here, we report on the CRISPR/Cas9 engineering of *S. suis* enolase mutants *eno^261^*, *eno^252/253/255^*, *eno^252/261^*, and *eno^434/435^* possessing amino acid substitutions at *in silico* predicted binding sites for Plg. As expected, amino acid substitutions in the predicted Plg binding sites reduced Plg and Pln binding to *S. suis* but did not affect bacterial growth *in vitro* compared to the wild-type strain. The binding of Plg to wild-type *S. suis* enhanced translocation across the human cerebral microvascular endothelial cell line hCMEC/D3 but not for the *eno* mutant strains tested. To our knowledge, this is the first study where predicted Plg-binding sites of enolase have been mutated to show altered Plg and Pln binding to the surface of *S. suis* and attenuation of translocation across an endothelial cell monolayer *in vitro*.

## Introduction

*Streptococcus suis* (*S. suis*) is a major porcine pathogen worldwide, that can be asymptomatically carried in palatine tonsils. In weaning piglets, it is a frequent cause of invasive disease commonly resulting in sepsis and rapid onset meningitis (Wertheim et al., 2009). *S. suis* is also an emerging zoonotic pathogen posing a risk to persons exposed to infected pigs or contaminated pork products (Ho et al., 2011; Segura et al., 2020). Pathogenic *S. suis* comprises 29 polysaccharide capsule serotypes, of which serotype 2 is the most frequently associated with invasive disease in pigs and humans (Perch et al., 1983; Nomoto et al., 2015; Tohya et al., 2017). To develop effective prevention and control strategies against *S. suis*, there is a need to increase our understanding of the molecular mechanisms that are critical for virulence (Segura et al., 2017).

Bacterial translocation across the blood-brain barrier (BBB) is considered as the most critical step in the pathogenesis of *S. suis* meningitis (Gottschalk and Segura, 2000). The BBB, the barrier separating the central nervous system (CNS) blood and brain parenchyma, consists of brain microvascular endothelial cells (BMEC) with tight junctions and interacting pericytes and astrocytes (Coureuil et al., 2017). *S. suis* has been reported to adhere to but rarely invade human immortalized BMEC (Charland et al., 2000). Later, Rui et al. showed translocation of *S. suis* across BMEC monolayers and proposed an important role for the serine threonine kinase in disruption of tight junctions (Rui et al., 2018). Additionally, binding of human fibrinogen to *S. suis* virulence-associated protein muramidase-released protein (MRP) has been reported to disrupt the adherens junction protein p120-catenin in human cerebral microvascular endothelial cells (hCMEC/D3) to facilitate paracellular translocation (Wang et al., 2015).

The extracellular matrix (ECM) network of proteins such as collagen, laminin and fibronectin is in an important structural and biochemical component of endothelial and epithelial barriers, but some pathogens can degrade ECM components with bacterial proteases or by sequestering proteins of the host fibrinolytic system such as plasminogen (Plg) and plasmin (Pln) which are present in the bloodstream (Sanderson-Smith et al., 2012; Law et al., 2013; Parmer and Miles, 2013; Hammerschmidt et al., 2019). Immobilization of Plg to the surface or receptor enables it to be efficiently converted to Pln by tissue plasminogen activator (tPA) or urokinase plasminogen activator (uPA). The normal function of Pln is to degrade fibrin clots, connective tissues, and ECM proteins in response to tissue injury. However, the hijacking of Plg/Pln system by bacterial pathogens is proposed to facilitate transmigration across host epithelia/endothelia and spreading in tissues due to the proteolysis of ECM proteins (Ploplis and Castellino, 2005).

Enolase is an essential intracellular enzyme converting glucose into pyruvate and generating energy in the form of ATP and NADH in the process. It is also known as a “moonlighting” protein because it has been shown to have an auxiliary function in binding Plg on the surface of pathogenic bacteria and mammalian cells (Bergmann et al., 2003; Jones and Holt, 2007; Attali et al., 2008a, Attali et al., 2008b; Magalhaes et al., 2013; Parmer and Miles, 2013; Cork et al., 2015). Purified enolase from *S. suis* has been shown to bind to Plg *in vitro* (Esgleas et al., 2008; Gottschalk et al., 2010). *S. suis* enolase has also been detected on the bacterial surface using immunoelectron microscopy and immunofluorescence assays (Feng et al., 2009). However, the essentiality of enolase prohibits the generation of deletion mutants to unequivocally demonstrate its role in Plg binding and transmigration across host cell barriers.

To investigate the interaction between bacterial enolase and host Plg, previous studies measured Plg binding to recombinant enolase proteins from *S. pneumoniae* and *S. pyogenes* containing substitutions at predicted Plg-binding sites (Bergmann et al., 2005a, Bergmann et al., 2001; Cork et al., 2015). The protein crystal structure of recombinant enolase from Group A Streptococcus (GAS), *S. pneumoniae*, and *S. suis* consists of an octamer formed from a tetramer of homodimers (Ehinger et al., 2004; Cork et al., 2009; Lu et al., 2012; Cork et al., 2015). In both *S. pneumoniae* and *S. pyogenes*, Plg binding assays using purified site-directed mutant enolase proteins identified two binding sites per monomer: an internal binding motif FYDKERKVY (amino acid residues crucial for Plg binding are bold and underlined), and a C-terminal double-lysine residues binding motif (Derbise et al., 2004; Bergmann et al., 2005b, Bergmann et al., 2003; Cork et al., 2009). *Streptococcus mutans* has only 5 amino acids in common with the internal binding motif of *S. pneumoniae* FYDNG**VY, of which only the aspartate required for Plg binding is conserved. Thus, only the C-terminal binding motif is considered critical for Plg binding to *S. mutans* enolase (Bergmann et al., 2003; Ehinger et al., 2004; Jones and Holt, 2007). In enolases of the non-pathogenic Gram-positive bacteria *Bifidobacterium lactis* and *Lactobacillus plantarum*, a similar internal binding motif was suggested as potential Plg-binding site (Candela et al., 2009; Vastano et al., 2013).

Although experiments with anti-enolase antibodies have demonstrated the importance of enolase for *S. suis* adherence and invasion of porcine brain endothelial cells (Esgleas et al., 2008), there remains a need to demonstrate the contribution of enolase to Plg and Pln binding to intact *S. suis* as well as the impact of reduced Plg binding on translocation of *S. suis* across the BBB. In this study, we predicted four potential Plg-binding sites of *S. suis* enolase via multiple sequence alignment (Wunsch and Needleman, 1970) and protein superimposition (Sippl, 1996). To validate predicted Plg-binding sites in *S. suis* enolase, we used recently described vectors for CRISPR/Cas9-mediated genome editing in *S. suis* (Gussak et al., 2023) to generate four site-substituted *S. suis* enolase mutant strains. All enolase mutants showed similar growth as wild-type *S. suis* but reduced Plg-binding ability, suggesting that all four predicted sites contribute to Plg-binding. Finally, we found that Plg binding to *S. suis* enolase facilitates *S. suis* translocation across hCMEC/D3 monolayers, an *in vitro* model of the BBB. Our results reveal binding sites required for Plg binding to *S. suis* enolase and the role of Plg binding by enolase in promoting bacterial translocation across the BBB.

## Materials and methods

### Multiple sequence alignment analysis and sequence identity analysis

The amino acid sequences of enolase proteins of *S. suis* (SSU1320), *S. pneumoniae* (SPD_1012), *S. pyogenes* (Spy0731), *B. lactis* (AAZ22544.1), *L. plantarum* (JDM1_0656), *and S. mutans* (SMU.1247) were aligned using ClustalX2 (Larkin et al., 2007). Protein structural information is more reliable than the corresponding amino acid sequence for inferring the function of protein as it changes less than the protein sequence during evolution (Rost, 1999). Therefore, we predicted *S. suis* P1/7 enolase secondary structure with the sequence alignment outputs using ESPript 3.0 (Robert and Gouet, 2014) to investigate homologous motifs among different enolase proteins. The enolase gene sequence identity of various streptococcal strains was analyzed using the Nucleotide Basic Local Alignment Search Tool (BLASTn).

### Superimposition of enolase proteins

The basic purpose of protein superimposition is to develop an optimal conformational alignment of protein structures with minimized root-mean-square-deviation (RMSD) of alpha-carbon (Cα) atom pairs and to analyze the conservation of protein folds across the aligned proteins. The tertiary structure of *S. suis* P1/7 enolase (SSU1320) was predicted based on the enolase gene coding sequence and using the resolved three-dimensional structure of *S. suis* strain 05ZYH33 enolase (PDB entry *4ewj*) as the template through the Robetta server (Lu et al., 2012). *S. pneumoniae* enolase and *L. plantarum* enolase, of which Plg-binding motifs had been determined (Bergmann et al., 2003; Vastano et al., 2013), were selected as structural templates to respectively perform pairwise structural rigid-body superimposition with predicted *S. suis* enolase three-dimensional structure in PyMoL software (The PyMOL Molecular Graphics System, Version 2.5.2 Schrödinger, LLC). In each superimposition, *S. suis* enolase was computationally rotated and transformed to align to the template enolase with minimized RMSD of C_α_ atom pairs. A typical RMSD value for homologous proteins was proposed as 3 Å (Chothia and Lesk, 1986). Lower RMSD values predict higher similarity between two proteins. Protein superimposition outputs containing specific homologous amino acid residues or motifs were generated by PyMoL software and exported as images. Plg-binding motifs of *S. pneumoniae* enolase and *L. plantarum* enolase were used to predict Plg-binding motifs of *S. suis* enolase *in silico* because of the structural similarity. All PyMoL commands needed during the superimposition could be found in the PyMoL Command Reference web (https://pymol.org/pymol-command-ref.html).

### Generation of site-directed point mutations in *S. suis* enolase

Four *eno* mutant strains were generated by the CRISPR/Cas9-mediated genome editing. This involved four steps: (i) the construction of gRNA-Cas9 co-expression plasmid, (ii) the construction of the DNA repair template, (iii) the transformation of *S. suis*, and (iv) validation of the gene editing. Guide RNAs (gRNAs) which form an alternative to the crRNA:tracrRNA were designed in Benchling (Biology Software. Retrieved from https://benchling.com) and potential off-target scores were checked using CRISPR RGEN Tools (http://www.rgenome.net/cas-designer/) (Bae et al., 2014; Park et al., 2015). The best gRNA sequence was cloned into plasmid pSS by annealing of two oligonucleotides containing overhangs that anneal to the BsaI digested vector. Plasmid pSS encodes Cas9 and a chloramphenicol-resistance gene (Gussak et al., 2023). Details of thermocycler conditions for generating gRNA and inserting gRNA into pSS are included in the **Supplementary Material**. Four different repair templates were generated with the mutated Plg-binding sites with approximately 1000 bp of homologous sequence upstream and downstream the *eno* gene and cloned into plasmid pUC57 which encodes a kanamycin-resistance gene (Gussak et al., 2023). Detailed sequences of all DNA fragments included in the repair templates can be found in **Supplementary Table S1** and corresponding thermocycler conditions are included in the **Supplementary Material**. At predicted Plg-binding sites Lys-261, Lys-252/Glu-253/Lys-255, Lys252/Lys-261, and Lys-434/435, the lysine residues were substituted by alanine and glutamic acid by glycine. For remaining residues in the gRNA complementary region, we replaced codons with amino acids representing same proteins so that the repair template could escape the Cas9-mediated double stranded DNA breaks.

*Escherichia coli* DH5α cells (NEB, C2987H) were transformed with the shuttle vectors harboring gRNA-Cas9 or repair templates and were grown in the Luria Bertani Broth (LB) with the required antibiotics: 25 µg/ml of chloramphenicol or 50 µg/mL of Kanamycin, respectively. Finally, we transformed co-expression vectors containing the respective gRNAs and corresponding repair templates into wild-type *S. suis*, as previously described (Zaccaria et al., 2016) with slight modification. Transformed *S. suis* strains were grown overnight at 37 ˚C with 5% CO_2_. The overnight culture was diluted 1:40 with fresh Todd Hewitt Broth (THB; Difco Laboratories, Detroit, MI, The United States) with 0.2% yeast extract (THY) medium and incubated at 37 ˚C until the optical density at 600 nm (OD_600nm_) was between 0.035 and 0.058. Aliquots of 100 µL of *S. suis* liquid culture were transferred into 1.5 mL Eppendorf Safe Lock Tubes™ and combined with 5 µL synthetic competence-inducing peptide solution (Zaccaria et al., 2016) (5 mM, GenScript), 1 µg of pSS/gRNA-Cas9, and 2 µg of DNA repair template. After 2 h of incubation at 37 ˚C with 5% CO_2_, the mixture was spread onto THY agar medium containing chloramphenicol at a final concentration of 5 µg/mL.

### Bacterial culture conditions and cell line

*S. suis* serotype 2 strain P1/7 and the four isogenic strains carrying the mutations in the predicted Plg-binding sites *eno^261^*, *eno^252/253/255^*, *eno^252/261^*, and *eno^434/435^* were grown overnight in 10 mL THY at 37 ˚C with 5% CO_2_. After overnight culture, the bacterial suspension was centrifuged at 4000 rpm for 10 min, resuspended in 10 mL fresh THY medium, measured for OD_600nm_, and diluted to OD_600nm_ = 0.08. Subsequently, wild-type and *S. suis* enolase mutants were cultured an additional one hour to reach concentrations of about 3×10^8^ CFU/mL and used for growth and Plg binding experiments. *S. suis* strains were cryopreserved in 50% glycerol-THY at -80 ˚C.

The human cerebral microvascular endothelial cell line (hCMEC/D3) used as a model of the BBB was purchased from Merck (Cat.SCC066) and cultured in commercial EndoGRO™-MV Complete Media Kit (Milipore, Cat. # SCME004) supplemented with 1 ng/mL of FGF-2 (Cat.GF003) according to the manufacturer’s recommendations.

### Growth curves of wild-type and enolase mutant *S. suis*

Bacteria were cultured as described above and growth measured by OD_600nm_ using a spectrophotometer (Molecular Devices, CA). Measurements were recorded for three independent bacterial cultures at each timepoint. Additionally, colony forming units (CFU) of bacteria in batch culture were counted. At each timepoint, a 1:10 serial dilution of each batch culture in PBS was performed and 10 µL from each dilution was spread on THY agar media using triplicate plates for each timepoint. Plates were incubated overnight at 37 °C with 5% CO_2_ and colonies counted the next day. The average CFU/mL of the *S. suis* culture was calculated from three independent experiments for each timepoint.

### Cloning, expression, and purification of recombinant *S. suis* P1/7 enolase protein

Protein expression plasmid pNIC28-Bsa4 was used for expression of wild-type and mutant enolase as previously described with minor modification (Charalampos Aslanidis, 1990). The *eno* genes were amplified using the genomic DNA as template from wild-type *S. suis* or plasmids carrying the *eno^251^* homologous repair templates as described in the **Supplementary Material**. The PCR products were recovered after agarose gel electrophoresis, treated by T4 DNA polymerase and dCTP, and then combined with T4 DNA polymerase and dGTP-treated pNIC28-Bsa4. After 2 h of incubation at 22 ˚C, 5 µL of each pNIC-*eno* construct was transformed to chemically competent *E. coli* DH5α cells (NEB, C2987H) which were subsequently plated onto LB agar media containing kanamycin. The colonies containing the correct pNIC-*eno* constructs were identified by colony PCR and then cultured in fresh LB medium with 50 µg/mL of kanamycin at 37 ˚C overnight with shaking at 220 rpm. The pNIC-*eno* plasmids were isolated using the Qiagen QIAprep^®^ Spin Miniprep Kit (Qiagen) according to the manufacturer’s instructions. The concentration of plasmid DNA was measured by DS-11 spectrophotometer (Denovix, Wilmington, DE, The United States). Constructs was verified by sequencing the purified DNA plasmid (Eurofins Genomics).

For protein expression, Rosetta (DE3) cells were transformed with the plasmids of wild-type enolase and *eno^251^* mutant by heat shock. After overnight culture, transformed Rosetta cells were grown in LB with 30 µg/mL of kanamycin and 40 µg/mL of chloramphenicol at 2 L scale. Bacterial cultures with an OD_600nm_ of 0.6 were induced by isopropyl-β-D-thiogalactopyranoside (IPTG) at a final concentration of 0.4 mM during overnight growth at 18 ˚C. Bacterial cells were harvested by centrifugation for 15 min at 4000 rpm and resuspended in 20 mL MiliQ water and then stored at -20 ˚C until further use. Resuspended bacterial cells were thawed and transferred in lysis buffer (25 mM Tris, 200 mM NaCl, 1 mM TCEP, pH 8.0). Cells were lysed by sonication: 125 s-on and 10 s-off on ice with an output power setting of 60%. Cell debris and insoluble proteins were removed by centrifugation for 30 min at 21,000 rpm at 4 ˚C, while the supernatant was loaded onto a column containing 3 mL Nickel beads and equilibrated with wash buffer (25 mM Tris, 200 mM NaCl, 1 mM TCEP, 20 mM imidazole, pH 8.0). The Nickel beads were washed with 20 mL wash buffer to elute unbound proteins and the his-tagged protein was eluted with 6 mL elution buffer (25 mM Tris, 200 mM NaCl, 1 mM TCEP, 200 mM imidazole, pH 8.0). All fractions of the eluate were analyzed by sodium dodecyl sulphate polyacrylamide gel electrophoresis (SDS-PAGE). For both wild-type and *eno^251^* enolase proteins, the first elution fraction was used for further purification using size-exclusion chromatography. The recovered proteins were incubated with 50 µL of 6 mg/mL his-TEV protease, transferred to a dialysis membrane and dialyzed overnight at 4 ˚C to remove the imidazole. Nickle beads were then used to remove the histone affinity tag essentially using the same procedure described above. Proteins were concentrated by Amicon Ultra-15 concentrator with 30,000 Da cut-off filters to a final volume of 1 mL and injected into a S200 16/60 gel filtration column, equilibrated in PBS. Fractions were collected and chromatograms were recorded. Aliquots of the fractionated proteins were prepared in PBS and snap-frozen in liquid nitrogen before storage at -80 ˚C. The final concentrations of purified soluble wild-type and *eno^251^* proteins in PBS were 2.5 mg/mL and 2.6 mg/mL, respectively.

### Measurement of enolase activity

To measure the activity of wild-type and *eno^251^* enolase, the conversion of 2-phospho-D-glycerate (2-PG) to phosphoenolpyruvate (PEP) by purified enolase protein was measured, as previously described (Pancholi and Fischetti, 1998). Briefly, different concentrations of 2-PG (0.5, 1, 2.5, 3, and 6 mM) were incubated with 5 µg of purified enolase protein at 37 ˚C for 3 min in HEPES/NaOH buffer (100 mM HEPES, 10 mM MgCl_2_, 7.7 mM KCl; pH 7.0) with a final volume of 750 µL. The enolase activity was recorded as the rate of change in the absorbance at 240 nm with a 5-s interval for a period of 3 min. To calculate the released rate of PEP, the Lambert-Beer formula (*A* = α_m_ *c*l) was used (*α_m_*: the extinction coefficient for PEP, 1.164×10^-3^ M^-1^). The relation between the concentration of 2-PG (*S*) and rate of PEP formation (*v*) was presented in Michaelis-Menten plot according to the equation *v* = d[P]/dt = V_max_ × [*S*] × (K_M_+[*S*])^-1^. V_max_ represented the maximum rate reached by the reaction at saturating substrate concentration and a given enzyme concentration. K_M_ was equal to the substrate concentration when the rate of product formation was half of V_max_. The reciprocal of both sides of the Michaelis-Menten equation was used to Lineweaver-Burk plot. The kinetic coefficients, K_M_ and V_max_, were calculated from the intercepts and slopes of the Lineweaver-Burk plot (Lineweaver and Burk, 1934).

### Plasminogen binding and plasmin activity assay

Wild-type and four *S. suis* enolase mutants were cultured in 10 mL THY medium overnight at 37 ˚C and centrifuged at 4000 rpm for 10 min. Bacterial pellets were resuspended in 10 mL fresh THY medium and the OD_600nm_ was measured. All bacterial suspensions were diluted to OD_600nm_ of 0.08 in 10 mL THY and subsequently subcultured for 1 h at 37 ˚C with 5% CO_2_ to obtain expected final concentrations for all strains based on the growth curve information. After subculturing, all cultures were pelleted and resuspended with PBS to the concentration of 1×10^9^ CFU/mL. Using these concentrated bacterial solutions, Pln activity assays were performed as previously described (Bergmann et al., 2005b, Vastano et al., 2013; Buscetta et al., 2016) with minor modifications. Briefly, 500 µL of bacterial suspension (5×10^8^ CFU *S. suis*) was incubated with 10 µL of human Plg (1 mg/mL, Sigma-Aldrich) in the presence or absence of 5.6 µL of recombinant human tPA (42.8 KIU/mL, abcam) for 2 h at 37 ˚C. Bacteria incubated with 20 µL of human Pln (0.3 units/mL, Sigma-Aldrich) for 2 h was used as positive control. After incubation, bacteria were washed with 600 µL of PBS to remove unbound Plg and then resuspended in 400 µL of chromogenic substrate D-Val-Leu-Lys-p-nitroanilide (VALY) (400 µM, Sigma-Aldrich). Absorbance at 405 nm was measured soon after addition of VALY but before resuspension (A_t0_). After 18 h of incubation at 37 ˚C with 5% CO_2_, bacteria were pelleted and the absorbance of 100 µL of the supernatant was measured at 405 nm (A_t18_) in a 96-microtiter plate. The activity of *S. suis*-bound Pln was calculated as ΔAbsorbance= A_t18_-A_t0_. As a negative control, bacteria were not incubated with Plg and only with tPA. The blank control was bacteria alone which were not incubated with Plg or tPA. All data were normalized relative to the blank control.

To determine the relation between Plg-binding ability and Pln activity of all *S. suis* strains in this study, a Pln activity standard curve was generated using the Plasmin Activity Assay Kit (ab204728) by plotting the rate of Relative Fluorescence Units (RFU) increase (ΔRFU/h) as a function of the amount of Pln (µg). As the initial amount of Plg added was the same for all strains, the Plg binding % reflects the Plg binding ability of wild-type and mutant strains of *S. suis*.

### Translocation assay

The hCMEC/D3 cell line was grown to confluency on collagen type I pre-coated 24-well plate Transwell inserts (Corning, Cat.353096). For each insert, 500 µL of cell suspension with the concentration of 3×10^4^ cells/mL in EndoGRO™-MV Complete medium was seeded, which yielded a density of 5×10^4^ cell/cm^2^. The wild-type and enolase mutant *S. suis* were diluted accordingly in EndoGRO™-MV Complete medium to a Multiplicity of Infection (MOI) of 10 bacteria per hCMEC/D3 cell. 500 µL of bacterial suspension with or without Plg plus tPA was inoculated in the upper chamber of inserts and 1.5 mL of pre-warmed EndoGRO™-MV Complete medium was added to the lower chamber of each well. 10 µL of 10 mM EDTA was added to the insert as a positive control for tight junction disruption, while the empty insert (without cells) was used as the blank for trans-endothelial electrical resistance (TEER) measurements. After 3 hours, all inserts were moved to a new 24-well plate containing 1.5 mL pre-warmed medium and TEER values were measured again. The lower chamber medium from the original plates were collected and serially diluted with PBS to enumerate translocated bacteria in each well. The translocation ratio was calculated after 3 hours as the bacterial CFU in the lower chamber divided by the bacterial CFU in the inoculum added to the upper chamber.

### Statistical analysis

At least three independent experiments were performed in each study and triplicate samples used for the measurements. Graphs were created using GraphPad Prism software version 9 (GraphPad Software). Results were presented as means ± standard deviations (SD). Student’s t test, One-way ANOVA, and Two-way ANOVA were implemented where appropriate to analyze the differences between groups in different experiments. A *P* value of < 0.01 was considered statistically significant.

## Results

### Sequence alignment and gene identity of *S. suis* enolase

**Figure 1:**
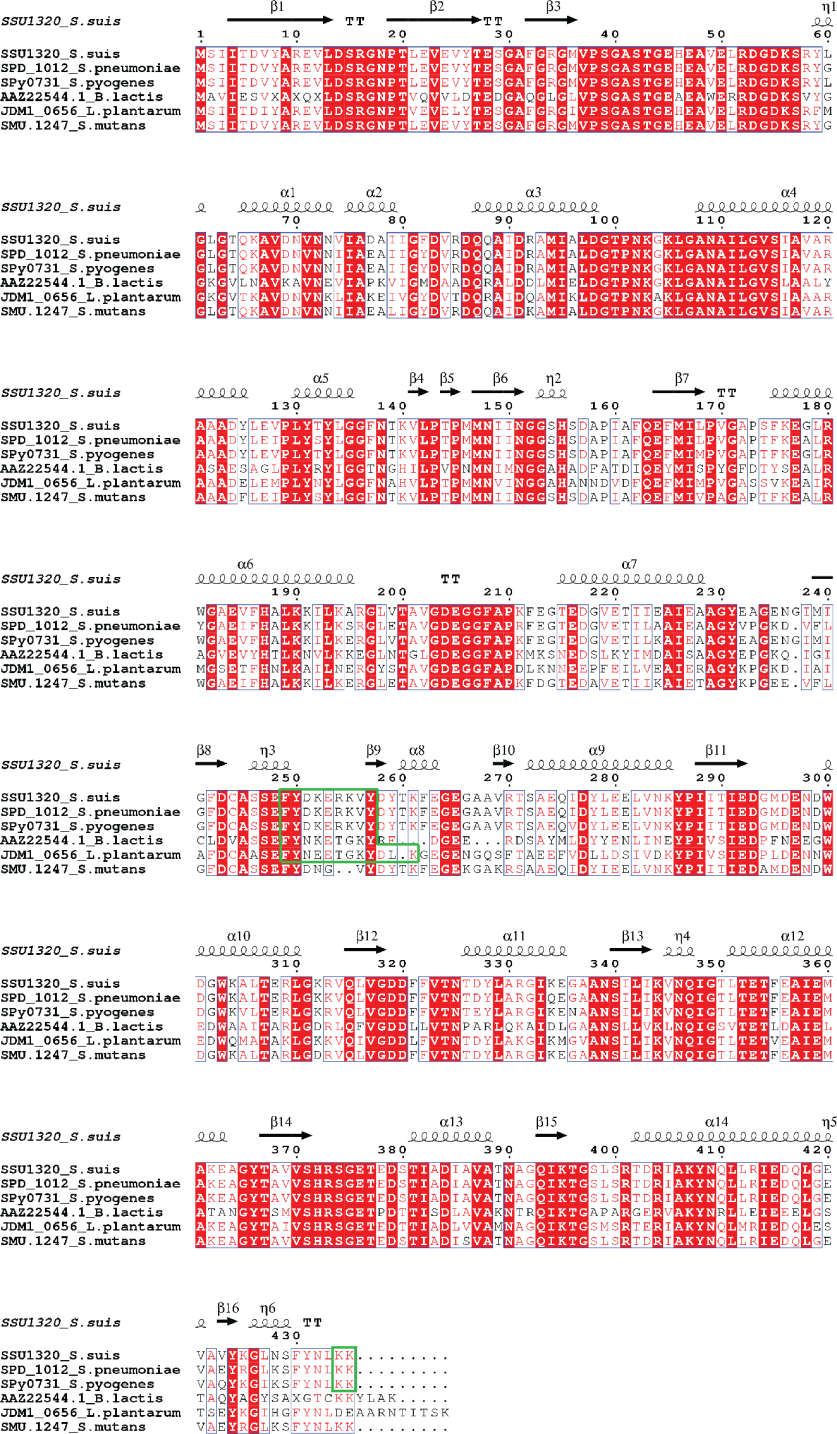
Multiple Sequence Alignment of amino acid sequences of full-length enolase proteins from *S. suis* (SSU1320), *S. pneumoniae* (SPD_1012), *S. pyogenes* (Spy0731), *B. lactis* (AAZ22544.1), *L. plantarum* (JDM1_0656), and *S. mutans* (SMU.1247). Identical residues are depicted in white letters with red background, while similar residues are in red letters with white background (similar residues are those having similar side-chain electrical charge and polarity). The predicted secondary structures of *S. suis* enolase regions are displayed above the corresponding aligned regions. α represents α-helix, β represents β-sheet, η represents coil, and T represents turn. Putative Plg-binding sites are indicated by the green boxes.

BLASTn search results showed that the enolase gene (*eno*) is present in all *S. suis* strains, with 98.62% to 100% nucleotide sequence identity (**Supplementary Table S2**), corroborating previous reports that *eno* is highly conserved in *S. suis* (Zhang et al., 2000; Morgulis et al., 2008). Sequences of enolase proteins from *S. pneumoniae*, *S. pyogenes*, *B. lactis*, *L. plantarum*, and *S. mutans* that have been reported to bind to Plg via specific Plg-binding motifs were aligned with the enolase amino acid sequence from the virulent *S. suis* strain P1/7 to reveal the conservation of proposed Plg-binding sites across all species (**Figure 1**).

### Predicted plasminogen-binding sites in *S. suis* enolase

**Figure 2:**
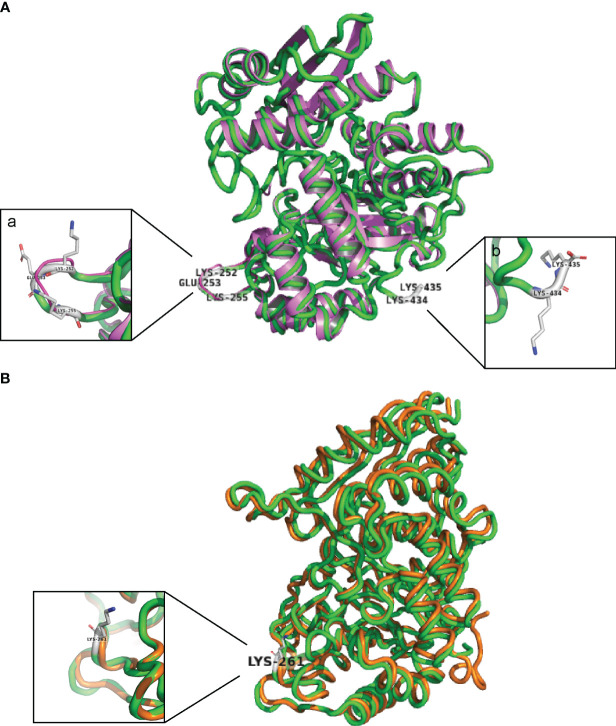
*S. suis* enolase protein superimposition with *S. pneumoniae* and *L. plantarum* enolase. **(A)** Cartoon of the structural superimposition between *S. suis* P1/7 enolase (green) and *S. pneumoniae* enolase (violet). a: Lys-252, Glu-253, and Lys-255 on *S. suis* enolase are highlighted with gray sticks; b: Lys-434 and Lys-435 are highlighted with gray sticks. **(B)** Superimposition of *S. suis* enolase (Green) and *L. plantarum* enolase (Orange). Lys-261 is indicated and shown in the inset.

To identify potential Plg-binding sites in *S. suis* enolase, the predicted tertiary structure was superimposed on the corresponding structures of *S. pneumoniae* enolase and *L. plantarum* enolase, for which Plg-binding motifs have been reported (Bergmann et al., 2003; Ehinger et al., 2004; Bergmann et al., 2005b, Vastano et al., 2013) (**Figure 2**). The predicted tertiary structure of *S. suis* enolase was similar to the crystal structure of *S. pneumoniae* enolase (**Figure 2A**). The RMSD (see Methods) of Cα atoms in the two superimposed proteins was 0.269 Å, which is lower than 3 Å, the value considered as threshold to delimit homologous proteins (Chothia and Lesk, 1986), suggesting the structure of both proteins is highly conserved. The pneumococcal Plg-binding site FYDKERKVY (in bold the amino acid residues crucial for Plg binding) is identical in *S. suis* and located with an RMSD of only 0.699 Å in the superimposed proteins. Four amino acids of *S. pneumoniae* enolase previously proposed to interact with Plg, Asp-250, Lys-251, Glu-252, and Lys-254, showed orthologous residues Asp-251, Lys-252, Glu-253, and Lys-255 in *S. suis* enolase (Bergmann et al., 2003; Ehinger et al., 2004) (**Figure 2A**-a). Although the C-terminal double-lysine residues of *S. suis* and *S. pneumoniae* enolases aligned with an RMSD value of 0.005 Å, site-directed mutagenesis of these lysine residues in *S. pneumoniae eno* gene did not impair Plg-binding, suggesting that it might not be a universal Plg-binding site (Bergmann et al., 2005b, Bergmann et al., 2003; Ehinger et al., 2004). Therefore, we hypothesized that Lys-434 and Lys-435 were also less likely to be a Plg-binding site in *S. suis* (**Figure 2A**-b). Our results disproved this hypothesis as described in the sections below.

Superimposition of the predicted tertiary structure of *S. suis* enolase with the crystal structure of *L. plantarum* enolase yielded a RMSD value of 0.177 Å, showing that the tertiary structure of *L. plantarum* enolase was more like *S. suis* than *S. pneumoniae* enolase (**Figure 2B**). The region from residue 249 to residue 261 with sequence FYDKERKVYDYTK in *S. suis* enolase and the region from residue 248 to residue 259 on *L. plantarum* enolase were closely aligned in the superimposed protein structures, with a RMSD value of 0.212 Å. Because Lys-259 has been proposed in protein as an effective Plg-binding site of *L. plantarum* enolase (Vastano et al., 2013), and this protein appears highly similar to *S. suis* enolase, we hypothesized that *S. suis* enolase Lys-261 might also contribute to Plg-binding (**Figure 2B**).

### Mutation of plasminogen-binding sites in *S. suis* enolase

**Figure 3:**
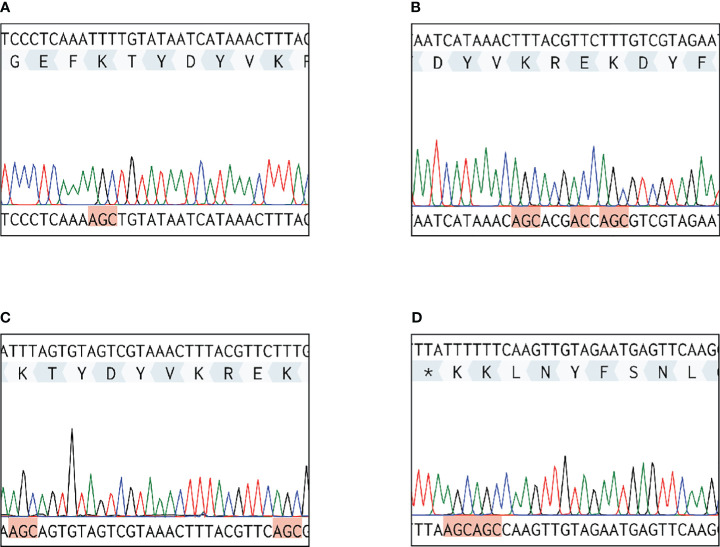
Sequencing results of DNA extracted from four *S. suis* enolase mutants. Each red highlighted frame represents a substituted amino acid. **(A)** *eno^261^* mutant. Lys-261 (TTT) is replaced by alanine (GCT). **(B)** *eno^252/253/255^* mutant. Lys-252/Glu-253/Lys-255 are replaced by alanine, glycine (GGT), and alanine, respectively. **(C)** *eno^252/261^* mutant. Lys-252/Lys-261 are replaced by alanine. **(D)** *eno^434/435^* mutant. Lys-434/Lys-435 are replaced by alanine. * Represents the terminate DNA codon (TTA).

**Figure 4:**
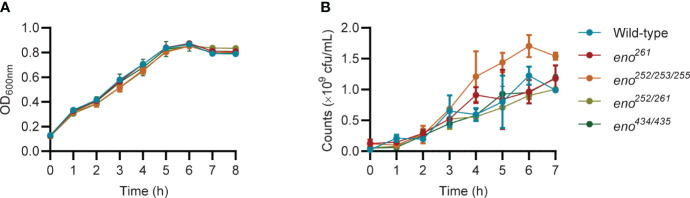
Growth curves of wild-type and four enolase mutants under static conditions. **(A)** All strains are monitored every hour by measuring OD_600nm_. **(B)** All strains are enumerated over time. Unpaired t-test is performed to compare growth of *eno^252/253/255^* to the wild-type strain across all timepoints for triplicates samples from three independent experiments.

The five putative Plg-binding sites encoded by the *S. suis eno* gene were modified by CRISPR/Cas9 gene editing to introduce amino acid substitutions predicted to affect binding. The substitution of Asp-251 (*eno^251^)* was not possible. Four obtained enolase mutant *S. suis* strains (*eno^261^*, *eno^252/253/255^*, *eno^252/261^*, and *eno^434/435^*) were verified by sequencing and their growth were compared in THY (**Figures 3**, **4A**). Plate counting of bacteria showed that the wild-type and all four enolase mutant strains remained in lag phase for about 2 hours, entered exponential phase after about 2 hours and stationary phase after around 6 hours (**Figure 4B**). Strains *eno^261^*, *eno^252/261^*, and *eno^434/435^* reached approximately 1 × 10^9^ CFU/mL after 6 h growth which was similar to wild-type at 6 h. Although *eno^252/253/255^* appeared to grow faster than other strains reaching a density of 1.7 × 10^9^ CFU/mL after 6 h (*P* = 0.003; three independent experiments), nonparametric multiple unpaired t-test analysis did not find significant differences between growth of *eno^252/253/255^* and the wild-type strain across all timepoints, revealing that the site-directed substitutions on Lys-252/Glu-253/Lys-255 of *S. suis* enolase have no effect on bacterial growth.

### Enzyme kinetics of wild-type and mutant enolases

**Figure 5:**
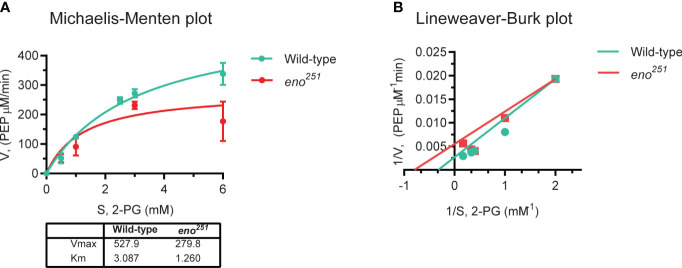
Enzymatic activity of purified wild-type enolase and *eno^251^* mutant protein. **(A)** Michaelis-Menten kinetics of two enolase proteins. The rate of enzymatic reaction is shown as the relation between the concentration of substrate (*S*) and rate of product formation (*v*). **(B)** Lineweaver-Burk plot of two enolase proteins. The equation is *y* = K_M_ × *x*/V_max_ + 1/V_max_, *y* is equal to *v* ^-1^ and *x* is equal to [*S*]^-1^. All data are collected from triplicates in two independent experiments.

As we failed to construct a viable *eno^251^* mutant strain, the recombinant wild-type and *eno^251^* mutant proteins were expressed and purified *in vitro* to detect whether the enzyme activity of *eno^251^* mutant protein was different from wild-type enolase. 2-PG-to-PEP conversion rates in the presence of different concentrations of substrate (2-PG: 0, 0.5, 1, 2.5, 3, 6 mM) were displayed in in a Michaelis-Menten plot **Figure 5A**, and the corresponding Lineweaver-Burk plot in **Figure 5B**. The values of K_M_ and V_max_ for wild-type enolase and *eno^251^* were determined to be 3.087 mM and 527.9 μM/min (wild-type), and 1.260 mM and 279.8 μM/min (*eno^251^*), respectively. The maximum rate of the enolase reaction (V_max_) was lower for *eno^251^* than for wild-type enolase in the presence of excess substrate while the substrate affinity (Km) of *eno^251^* was increased compared to wild-type. These results suggested that *eno^251^* has a higher affinity to the substrate 2-PG but a lower catalytic reaction rate. We speculate that this might be due to structural changes that affect the enzyme's active site or its ability to undergo conformational changes necessary for catalysis, explaining why we could not recover a viable *eno^251^* mutant.

### Plasminogen binding of wild-type and four *S. suis* enolase mutants

**Figure 6:**
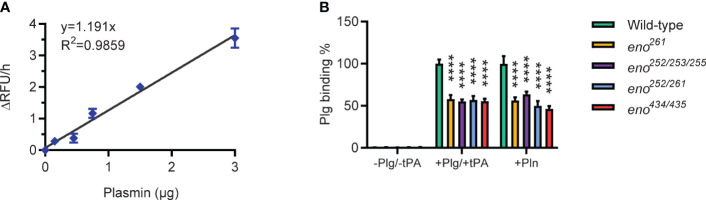
Plasminogen-binding ability of wild-type and *S. suis* enolase mutants. **(A)** Plasmin activity standard curve. Duplicates for each Pln concentration are used to generate the standard curve and derive the linear regression equation *y* = 1.191*x* with R^2 =^ 0.9859. **(B)** Plg binding ability. Plg binding % is calculated as the increase of absorbance of mutant divided by that of wild-type. -Plg/-tPA: *S. suis* is directly incubated with Pln substrate (VALY); +Plg/+tPA: *S. suis* is incubated with Plg and tPA; +Pln: *S. suis* is incubated with Pln. Statistical significance is calculated by two-way ANOVA. Error bars show the SD around the mean value for triplicates in three independent experiments. **** P < 0.0001.

We determined Plg-binding ability of *S. suis* by conversion of bound Plg to Pln with tPA and measuring Pln activity with a chromogenic substrate. A standard curve was generated with purified Pln to calculate the amount of Pln in the assay (**Figure 6A**). All four enolase mutants bound significantly less Plg than the wild-type *S. suis* strain (*P* < 0.01), showing that the amino acid substitutions indeed reduced Plg-binding (**Figure 6B**). The similar effect of different amino acid substitutions on bacterial Plg-binding ability suggested that all potential binding sites contribute to binding affinity. All identified Plg-binding sites likely contribute to Pln-binding because all four enolase mutants were observed to bind less Pln than wild-type *S. suis*.

### Translocation of wild-type and *S. suis* enolase mutants across endothelial cells

**Figure 7:**
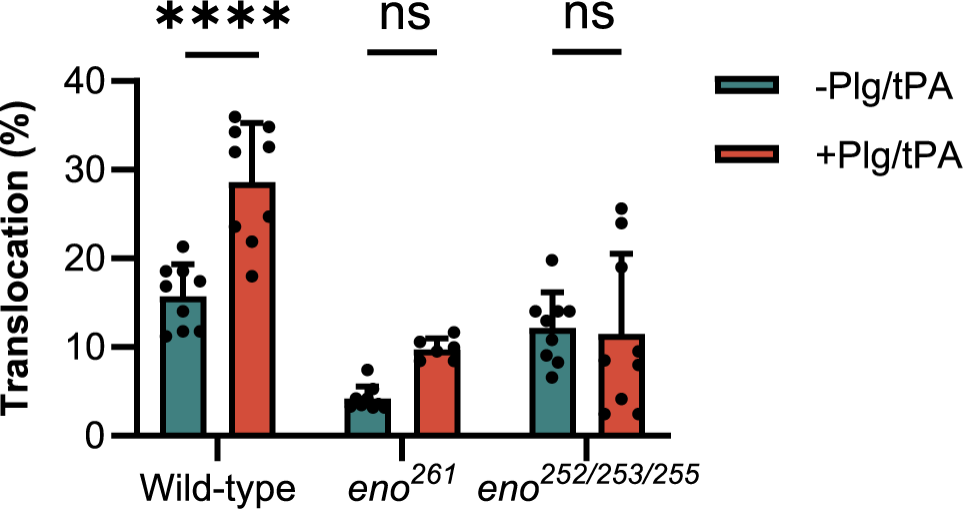
*S. suis* translocation assay. The translocation of mutants (*eno^261^* and *eno^252/253/255^*) is compared to that of wild-type *S. suis*. Same numbers of wild-type and mutant *S. suis* are inoculated in the upper chambers of Transwells containing hCMEC/D3 cells with or without adding Plg and tPA. After 3 h of incubation, medium in lower chambers is serially diluted and plated to enumerate translocated bacteria. The percentage translocation is calculated as the translocated CFU divided by inoculum CFU. Statistical significance is calculated by one-way ANOVA for all strains. Error bars represent the SD for triplicates in three independent experiments. -Plg/-tPA: without neither Plg nor tPA; +Plg/+tPA: with Plg and tPA. **** P < 0.0001: ns, not significant.

To investigate if Plg binding to *S. suis* enolase contributes to *S. suis* translocation across the BBB, we compared transmigration of the enolase mutants (*eno^261^* and *eno^252/253/255^*) with that wild-type *S. suis* across hCMEC/D3 cell monolayers. Before starting the experiment, the TEER of the hCMEC/D3 cells monolayer was measured using an epithelial Volt-Ohmmeter (EVOM3) device (World Precision Instruments, Europe) to ensure that all inserts contained intact monolayers with proper barrier function. Only inserts with TEER values higher than 100 Ω × cm^2^ were used for *S. suis* translocation assay (Santiago-Tirado et al., 2019) (**Supplementary Figure S1**). Log-phase wild-type and enolase mutants with human Plg plus tPA were applied to the upper chamber at a MOI of 10 bacteria per endothelial cell, while *S. suis* incubated with human Pln was regarded as the positive control. After 3 h of infection, the medium in the lower chamber was removed and dilutions were plated on THY agar to enumerate CFU of *S. suis* that had crossed the hCMEC/D3 monolayer. The translocation ratio was calculated as the ratio of translocated *S. suis* to the inoculum added to the upper chamber. The addition of Plg to wild-type *S. suis* significantly increased bacterial translocation across the BBB but had no effect on translocation of the *eno^252/253/255^* and *eno^261^* mutants (**Figure 7**). These results indicated that Plg-binding sites in enolase contribute to wt *S. suis* translocation across the BBB (**Figure 7**).

## Discussion

Binding of host Plg by Gram-positive bacteria and its subsequent conversion to Pln by plasminogen activator (PA) is considered important for the degradation of ECM and translocation of bacteria across epithelial and endothelial barriers during infection. Here, we predicted Plg-binding sites in *S. suis* enolase by multiple sequence alignments and protein tertiary structure superimposition of enolases protein structures from other bacteria for which Plg-binding sites have been investigated. Streptococcal enolases form octamers consisting of two types of protein-protein interfaces: a major interface involved in dimerization and a minor interface involved in the formation of tetrameric homodimers. In the octameric configuration, the internal Plg-binding motif FYDKERKVY identified in *S. pneumoniae* is present on the surface of enolase, while the proposed C-terminal Plg-binding motif is buried in the minor interface (Bhattacharya et al., 2012). Substituting residues in the minor interface leads not only to dissociation of tetramers, but also to impairment of the major interface. Therefore, mutations of the C-terminal double-lysine residues may lead to destabilization of both interfaces, which may change the structural integrity of enolase octamers (Karbassi et al., 2010).

Multiple *in silico* sequence alignments and protein superimpositions of *S. suis*-*S. pneumoniae* and *S. suis*-*L. plantarum* enolase were carried out to predict potential Plg-binding sites of the *S. suis* enolase. Five Plg-binding sites were predicted in *S. suis* enolase (K251A, K261A, K252A/E253G/K255A, K252A/K261A, and K434/K435A) (**Table 1**). We attempted to construct site-directed substitutions at all predicted Plg-binding sites in *S. suis* enolase. While we succeeded for *eno^261^*, *eno^252/253/255^*, *eno^252/261^*, and *eno^434/435^*, it was not possible for *eno^251^*. Plasminogen binding assays with the purified recombinant *eno^251^* protein revealed a higher binding affinity for its substrate 2-PG and a lower enzyme reaction rate compared to wild-type enolase. It is known that enolase is a critical glycolytic enzyme catalyzing reversible conversion of 2-PG to PEP. The reduced enzymatic activity of *eno^251^* might be the reason we could not recover this mutant strain. We speculated that substituting aspartic acid for alanine at residue 251 might have interrupted the major interface and thus destabilized the dimer, which is necessary to stabilize the catalytic sites of enolase (Kornblatt et al., 1998; Pal-Bhowmick et al., 2007; Karbassi et al., 2010). Putative catalytic sites in *S. suis* enolase consists of residues Glu164, Glu205, and Lys344, corresponding to the catalytic residues Glu164, Glu205, and Lys342 in the *S. pneumoniae* homologue (Lu et al., 2012). In *S. pyogenes*, two loop regions residues 152-159 and residues 245-266 together with residues 38-45 close the active site of enolase (Cork et al., 2015). The residue 251 is localized in the S6/H7 loop region exposed on the surface of the monomer of *S. suis* enolase where it is accessible to for interaction with other parts of the protein. The force stabilizing the enolase dimer comes from three inter-patch regions (1, 2, and 3) each containing 26 amino acids that stabilize the monomer interactions. Inter-patch 1 mainly localizes in the N-terminal domain, with Glu378, Thr402, and Asp403 localized at H11 and H12, respectively. The inter-patch 1 of one monomer contacts inter-patch 2 of the other monomer, and vice versa. Asp251 is localized between S6 and H7 and in proximity to other important residues Thr402, and Asp403 as part of eight large helices (H5-H12) sequentially surrounding the core strands (S4-S11) in *S. suis* enolase (Lu et al., 2012). Therefore, the substitution of residue 251 could be significant for the overall stability of the enolase dimer and potentially its function.

**Table 1:** Site-substitutions of potential Plg-binding sites in *S. suis* enolase.

	Internal motif	C-terminal motif
Wild-type	^249^FYDKERKVYDYTK^261^	^434^KK^435^
*eno^261^*	^249^FYDKERKVYDYTA ^261^	^434^KK^435^
*eno^252/253/255^*	^249^FYDAGRAVYDYTK^261^	^434^KK^435^
*eno^252/261^*	^249^FYDAERKVYDYTA ^261^	^434^KK^435^
*eno^434/435^*	^249^FYDKERKVYDYTK^261^	^434^ AA ^435^

Text with underlined represent substituted amino acids in corresponding enolase mutant strains.

All obtained enolase mutants displayed similar growth curves compared to wild-type *S. suis*, suggesting that site-specific substitutions at corresponding sites did not impact on the essential glycolytic activity of enolase and *S. suis* growth. All four mutants displayed significantly reduced Plg-binding ability compared to wild-type *S. suis*. Similarities in growth but differences in Plg-binding ability between wild-type and *S. suis* mutants reveal the multifunctionality of enolase protein, as suggested for other streptococci (Read et al., 1994; Bergmann et al., 2001, 2003, 2004; Castaldo et al., 2009; Ghosh et al., 2011; Bergmann et al., 2013; Fulde et al., 2013; Bao et al., 2014).

We found that Pln could also bind to wild-type *S. suis*, but the measured Pln activity was less than that obtained by first binding Plg and subsequent conversion to Pln by tPA. Plg can occur in three different conformations: the completely closed α-conformation, the semi-closed β-conformation, and the completely open γ-conformation (Rijken and Sakharov, 2001). Commonly, Plg exists in the closed α-conformation which has the highest binding affinity to receptor proteins such as histone H2B and amphoterin (Parkkinen and Rauvala, 1991; Herren et al., 2006). Conversely, Pln has an open conformation and lower binding affinity than Plg for target proteins, which probably explains the reduced amount of Pln bound on the surface of *S. suis* (Ponting et al., 1992; Mogues et al., 2004; Ploplis and Castellino, 2005). Pln-binding was diminished in all four *S. suis* mutants compared to wild-type strain. This indicated that all putative Plg-binding sites are likely Pln-binding sites in *S. suis* enolase. Lu et al. reported that purified K434A/K435A mutant enolase protein from *S. suis* 05ZYH33 strain increased Plg-binding ability compared to wild-type enolase protein via surface plasmon resonance measurements (Lu et al., 2012). On the contrary, our *eno^434/435^* mutant (in strain P1/7) bound less Plg compared to wild-type *S. suis*. Lu et al. only tested binding of Plg to purified protein rather than Plg binding to bacteria. Therefore, we speculate that these amino acid substitutions might have influenced the translocation of enolase to the bacterial cell surface, thereby reducing Plg-binding to our mutant strains.

To demonstrate the role of Plg in *S. suis* translocation across the *in vitro* BBB model, two enolase mutants *eno^261^*, *eno^252/253/255^* and the corresponding wild-type strain were incubated with Plg and tPA and added to the upper chamber of Transwell inserts containing a confluent hCMEC/D3 cell monolayer. We found that incubation of wild-type *S. suis* with Plg and tPA significantly increased translocation across hCMEC/D3 monolayers compared to the control (without Plg and tPA). In contrast, the translocation of *eno^261^* and *eno^252/253/255^* was not significantly increased by addition of Plg and tPA, likely due to their reduced Plg binding capacity (**Figure 7**). In the absence of Plg and tPA, translocation of *eno^261^* was significantly lower than that of wild-type *S. suis* (P = 0.0002), whereas there was no significant difference for *eno^252/253/255^* (P = 0.5890). This result suggested that *eno^252/253/255^* is a suitable enolase mutant for our research objective because of its impaired Plg-binding ability but consistent translocation compared with the wild-type *S. suis*. We also conducted adhesion and invasion assays of wild-type and *eno^252/253/255^* strain using hCMEC/D3 endothelial cell monolayers. Our findings did not show significant differences between wild-type and *eno^252/253/255^* for adherence (P = 0.9998) and invasion (P = 0.9773) without addition of Plg and tPA (**Supplementary Figure S2**), which confirmed that substitutions of residues 252/253/255 did not affect the *S. suis* interaction with hCMEC/D3. Additionally, there was no significant effect of adding Plg on adherence and invasion for the *eno^252/253/255^* mutant while addition of Plg significantly enhanced adhesion of the wild-type *S. suis* strain to hCMEC/D3 cells (**Supplementary Figure S2**). Our results further support previous conclusions that *S. suis* rarely invades human BMEC (Charland et al., 2000).

In conclusion, we applied an *in silico* and *in vitro* site-directed mutational approach to engineer amino acid substitutions in four candidate Plg-binding sites, namely K261A, K252A/E253G/K255A, K252A/K261A, and K434A/K435A, of *S. suis* enolase and validated that these sites were indeed involved in Plg- and Pln-binding to the surface of *S. suis*. We found that Plg binding to *S. suis* enolase and subsequent conversion to Pln facilitated *S. suis* translocation across the *in vitro* BBB model hCMEC/D3 cell line, although translocation still occurs in the absence of Plg. In this work, we identified critical Plg-binding sites in the *S. suis* enolase and demonstrated a contribution of Plg-enolase interaction to *S. suis* translocation using an *in vitro* model of the BBB.

## Data availability statement

The original contributions presented in the study are included in the article/**Supplementary Material**. Further inquiries can be directed to the corresponding author.

## Author contributions

TZ: Conceptualization, Data curation, Formal Analysis, Investigation, Methodology, Writing – original draft. AG: Methodology, Resources, Writing – review & editing. BH: Writing – review & editing. SB: Supervision, Writing – review & editing. PB: Conceptualization, Supervision, Writing – review & editing. JW: Conceptualization, Methodology, Project administration, Resources, Supervision, Writing – review & editing.
