# Supplementary material for: Identification of plasminogen-binding sites in Streptococcus suis enolase that contribute to bacterial translocation across the blood-brain barrier
Source: Front Cell Infect Microbiol. 2024 Feb 22;14:1356628. doi: 10.3389/fcimb.2024.1356628 (PMC10919400; doi:10.3389/fcimb.2024.1356628)
Supplement: Supplementary file 1 [file DataSheet_1.docx]

Supplementary Material

Identification of plasminogen-binding sites in *Streptococcus suis* enolase that contribute to bacterial translocation across the blood-brain barrier

**Tiantong Zhao, Alex Gussak, Bart van der Hee, Sylvia Brugman, Peter van Baarlen, Jerry M. Wells^*^**

*** Correspondence:** Jerry M. Wells: jerry.wells@wur.nl

# Supplementary Data

## Construction of gRNA-Cas9 co-expression plasmid

Briefly centrifuge the gRNA primers and suspend in 10 mM Tris to the concentration of 100 µM. Dilute gRNA primers to 10 µM by Nucle-free water. Prepare gRNA primer annealing master mix as follows:

| Components | Volume per 50 µL reaction (µL) |
| --- | --- |
| Forward primer | 5 |
| Reverse primer | 5 |
| Buffer TA (10 mM Tris + 50 mM NaCl + 1 mM DETA) | 40 |

Perform PCR program as follows:

| Step | PCR temp. (^o^C) | Time (min) |
| --- | --- | --- |
| Initial denaturation | 95 | 5 |
| Annealing and extension | Ramp down to 25 ^o^C at 1 ^o^C/min | - |

Ligate annealed double-strand gRNA oligos to the plasmid pSS containing Cas9 gene. Master mix preparation is shown below:

| Components | Volume per 25 µL reaction (µL) | Final concentration |
| --- | --- | --- |
| 10x T4 buffer | 2.5 | 1X |
| T4 DNA ligase (2000 Units/µL) | 0.5 | 1000 units |
| BsaI (20 Units/µL) | 1.5 | 30 units |
| pSS (220 ng/µL) | 0.35 | 75 ng/µL |
| gRNA oligos | 1 |  |
| Nucle-free water | 19.15 |  |

The PCR program is shown below:

| Step | PCR temp. (^o^C) | Time (min) | Cycles |
| --- | --- | --- | --- |
| Digestion | 37 | 5 | 60× |
| Ligation | 16 | 5 |  |
| Denaturation | 60 | 15 | 1× |
| Storage | 12 | ∞ | - |

## Construction of four *eno^mut^* repair templates

Two pairs of primers were designed to correspondingly amplify the upstream 1000 bp fragment and downstream 1000 bp fragment of enolase Plg-binding site mutation region. The preparation of master mix is shown like below:

| Components | Volume per 50 uL reaction (µL) | Concentration |
| --- | --- | --- |
| 5XQ5 Hot Start Reaction Buffer | 10 | 1× |
| 10 mM dNTPs | 1 | 200 μM |
| 10 μM Fw primer | 2.5 | 0.2-1 μM(0.5 μM) |
| 10 μM Re primer | 2.5 | 0.2-1 μM(0.5 μM) |
| Template DNA | 4 | <1000 ng |
| Q5 Hot Start DNA polymerase | 0.5 | 1× |
| Nucle-free water | 29.5 | 0.02 U/μL |

PCR program for amplifying upstream fragment and downstream fragment is shown:

| Step | PCR temp. (^o^C) | Time | Cycles |
| --- | --- | --- | --- |
| Initial denaturation | 98 | 30 s | 1× |
| Denaturation | 98 | 5 s | 34× |
| Annealing | 56 for upstream  61 for downstream | 10 s |  |
| Extension | 72 | 1 min 30 s |  |
| Final extension | 72 | 2 min | 1× |
| Storage | 12 | ∞ | - |

*Use NEB Tm calculator to calculate annealing temperature of Fw_US & Re_US and Fw_DS & Re_DS,, but we choose to decrease 2 ^o^C of each when conducting PCR program. It makes efficiency higher.

Run an agarose gel to check the outcomes of PCR. Subsequently, US and DS fragments were extracted from PCR products and measured the concentration by Nanodrop. The repair template was constructed by assembling US fragment, DS fragment, synthetic fragment (correspond to four eno-mutation, and pUC57 backbone. The PCR reaction was performed at 50 ^o^C for 60 min and stored at -20 ^o^C for subsequent *S. suis* transformation.

The PCR master mix was prepared as follow:

| Components | | Volume per 20 µL reaction (µL) |
| --- | --- | --- |
| Fragments mix | pUC57  US fragment  Synthetic fragment  DS fragment | Total 7.85 µL |
| NEBuilder HiFi DNA assembly master mix | | 10 µL |
| Nucle-free water | | 2.15 µL |

## Amplification of wild-type *eno* and *eno^251^* genes

The PCR program is shown below:

| Components | Volume per 50 uL reaction (µL) | Concentration |
| --- | --- | --- |
| 5XQ5 Hot Start Reaction Buffer | 10 | 1× |
| 10 mM dNTPs | 1 | 200 μM |
| 10 μM Fw primer | 2.5 | 0.2-1 μM(0.5 μM) |
| 10 μM Re primer | 2.5 | 0.2-1 μM(0.5 μM) |
| Genomic DNA for wild-type or  *eno^251^* plasmid for *eno^251^* | 4 | <1000 ng |
| Q5 Hot Start DNA polymerase | 0.5 | 1× |
| Nucle-free water | 29.5 | 0.02 U/ µL |

# Supplementary Figures


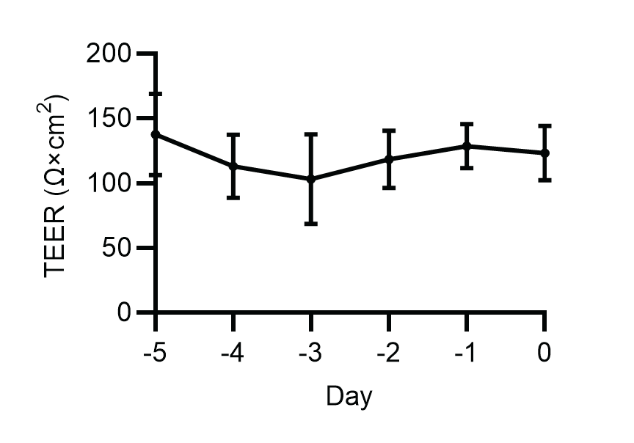


**Supplementary Figure 1.** TEER maintenance of hCMEC/D3 cell monolayer in prior to infection assays. Data presented as mean ± SD from three independent experiments (n=9).


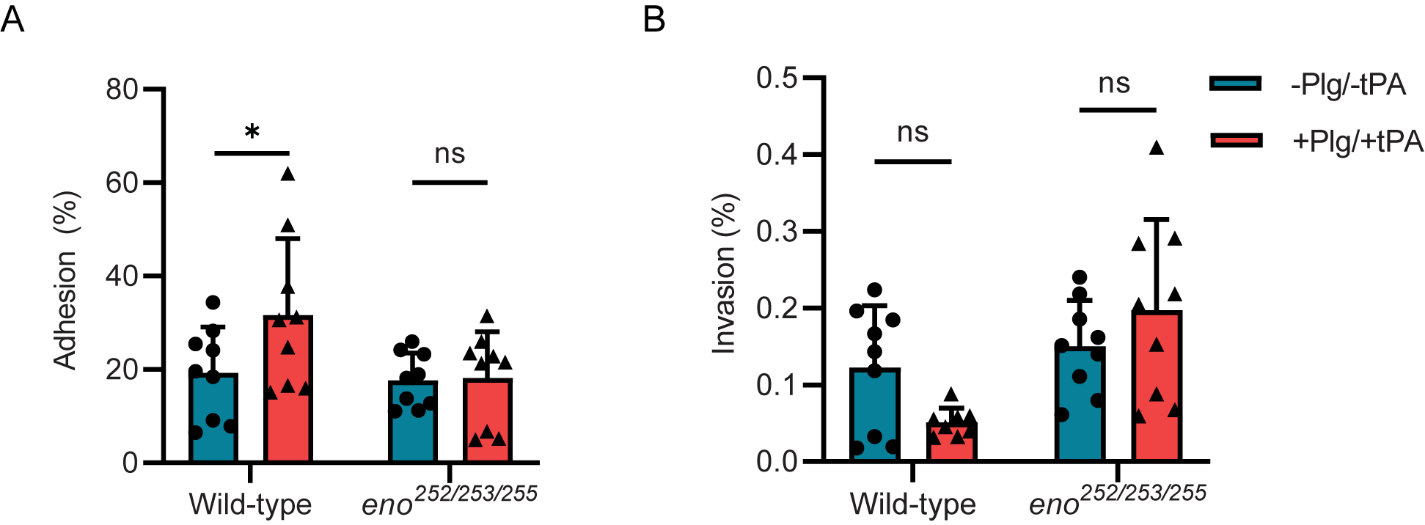


Supplementary Figure 2. *S. suis* adhesion and invasion assays. Log-phase wild-type and *eno^252/253/255^ S. suis* with or without Plg plus tPA were applied onto hCMEC/D3 monolayers with a multiplicity of infection (MOI) of 10 bacteria per endothelial cell. After 2 hours, wash cells three times with PBS to remove unadhered *S. suis* and add 500 µL of 0.05% Trypsin-EDTA following with 500 µL of 0.02% Triton-X100 to lyse cells and release adhered and intracellular *S. suis*. For invasion assay, 1-hour incubation of 100 ug/mL of gentamicin and 5 µg/mL penicillin G was followed to inactivate extracellular adhered *S. suis*. The error bars represent mean ± SD for triplicates in three independent experiments. -Plg/-tPA: without neither Plg nor tPA; +Plg/+tPA: with Plg and tPA.

# Supplementary Tables

Table S1 Oligonucleotides used in this study

| Oligos | Sequence (5’-3’) |
| --- | --- |
| guide RNA oligonucleotides | FW: tgatgcgtcaagagcgatcatagcg |
|  | RV: aaaccgctatgatcgctcttgacgc |
| Site-directed mutagenesis silent primers on repair template | FW: atctagggcaatcatggctcgatcgatagcttgttgatcacg |
|  | RV: gatcgagccatgattgccctagatggtactcctaacaaaggtaaattg |
| Synthetic *eno^251^* fragment | taccatcttcgatagtgatgattgggtatttgttaaccaattcttcaaggtagtcgatttgttctgcagatgtacgaacagcagcgccttcaccttcgaatttagtgtagtcgtaaactttacgttctttAGCgtagaattcagatgacgcacagtcgaaaccaatcatgatgccgttttcaccagcttcgtaaccagcagct |
| Synthetic *ENO^261^* fragment | taccatcttcgatagtgatgattgggtatttgttaaccaattcttcaaggtagtcgatttgttctgcagatgtacgaacagcagcgccttcaccttcgaaAGCagtgtagtcgtaaactttacgttcttttttgtagaattcagatgacgcacagtcgaaaccaatcatgatgccgttttcaccagcttcgtaaccagcagct |
| Synthetic *eno^252/253/255^* fragment | taccatcttcgatagtgatgattgggtatttgttaaccaattcttcaaggtagtcgatttgttctgcagatgtacgaacagcagcgccttcaccttcgaatttagtgtagtcgtaaacAGCacgACCAGCgtcgtagaattcagatgacgcacagtcgaaaccaatcatgatgccgttttcaccagcttcgtaaccagcagct |
| Synthetic *eno^252/261^* fragment | taccatcttcgatagtgatgattgggtatttgttaaccaattcttcaaggtagtcgatttgttctgcagatgtacgaacagcagcgccttcaccttcgaaAGCagtgtagtcgtaaactttacgttcAGCgtcgtagaattcagatgacgcacagtcgaaaccaatcatgatgccgttttcaccagcttcgtaaccagcagctt |
| Synthetic *eno^434/435^* fragment | gttcttactataaccctcgctatgctagctcagggataaaacagtctcccagactgttttaAGCAGCcaagttgtagaatgagttcaagcctttgtagactgcaacttcaccaagttgatcttcgatacgaagcaattggttgtatttagcgatacggtctgtacgtgacaatgaaccagtcttgatttggccagcgtta |
| Primers for upstream fragment of *eno^251^*, *eno^261^*, *eno^252/253/255^* and *eno^252/261^* | FW: aaggaatcacTTAGAATACTCTAAATGATACATGC |
|  | RV: CCAATCATCACTATCGAAG |
| Primers for downstream fragment of *eno^251^*, *eno^261^*, *eno^252/253/255^* and *eno^252/261^* | FW: TTCACCAGCTTCGTAACC |
|  | RV: cggattctgaAACTTATTTCCGACGACTTC |
| Primers for upstream fragment of *eno^434/435^* | FW: aaggaatcacACTTCTCTAGAGCATCTATTG |
|  | RV: GCATAGCGAGGGTTATAG |
| Primers for downstream fragment of *eno^434/435^* | FW: ACCAGTCTTGATTTGGCC |
|  | RV: cggattctgaAGTAATATAATGTCAATTATTACTGATGTTTAC |
| Primers for sequencing *eno^251^*, *eno^261^*, *eno^252/253/255^* and *eno^252/261^* mutation | FW: gcaccttctttgataccac |
|  | RV: caacgaagaccttctttg |
| Primers for sequencing *eno^434/435^* mutation | FW: gccccaaagtgccttatatc |
|  | RV: accaaatcggtactcttac |
| Primers for amplifying enolase coding gene | FW: TACTTCCAATCCatgtcaattattactgatgtttacg |
|  | RV: TATCCACCTTTACTGTCAttttttcaagttgtagaatgagttc |

Table S2 Sequence percent identity of the enolase gene in *S. suis*

| *S. suis* strains | Locus Tag | Gene Sequence Identity (%)* |
| --- | --- | --- |
| NCTC10234 | NCTC10234_01316 | 100 |
| CS100322 | CR541_07475 | 100 |
| LSM102 | A9494_06960 | 100 |
| SC19 | B9H01_07245 | 100 |
| SS2-1 | BVD85_07070 | 100 |
| ZY05719 | ZY05719_07125 | 100 |
| LSM178 | GRI05_07115 | 100 |
| 10 | SSU10_01323 | 100 |
| ISU2714 | DK876_07180 | 100 |
| ISU1606 | DK235_07185 | 100 |
| A7 | SSUA7_1335 | 100 |
| SS12 | SSU12_1387 | 100 |
| GZ1 | SSGZ1_1335 | 100 |
| 98HAH33 | SSU98_1513 | 100 |
| JS14 | SSUJS14_1470 | 99.92 |
| AH681 | CWI26_04450 | 99.69 |
| 13-00283-02 | SSU1300283_00791 | 99.54 |
| ISU2660 | DK878_04270 | 99.54 |
| ISU2414 | DK877_04385 | 99.54 |
| D9 | SSUD9_1510 | 99.54 |
| ST3 | SSUST3_1361 | 99.54 |
| TL13 | TL13_1329 | 99.46 |
| DAT299 | DAT299_13240 | 99.39 |
| SC183 | JZY07_08080 | 99.24 |
| D12 | SSUD12_1475 | 99.24 |
| SH1510 | DP111_03995 | 99.16 |
| NSUI060 | APQ97_06965 | 99.16 |
| GX69 | JZ789_07840 | 99.16 |
| YSJ17 | D2E16_04220 | 99.16 |
| 16085/3b | SSU16085_01472 | 99.16 |
| ISU2514 | DK875_07210 | 99.16 |
| HN136 | CWM22_04290 | 99.01 |
| AKJ18 | K6969_07865 | 98.93 |
| ISU2812 | A7J09_04970 | 98.85 |
| FJSM5 | K6974_04200 | 98.78 |
| 1112S | J1N58_04110 | 98.62 |

*Gene sequence identity was compared with the enolase gene coding sequence of *S. suis* strain P1/7.
